# Anterior rib giant cell tumor of bone mimicking a malignant chest-wall tumor: a case report

**DOI:** 10.1093/jscr/rjag872

**Published:** 2026-10-04

**Authors:** Mami Yamamoto, Masanori Ishida, Yuichi Ichikawa, Maki Tanigawa, Ai Enomoto, Akitoshi Kanagawa, Jun Nishida, Toshitaka Nagao, Kazuhiro Saito

**Affiliations:** Department of Radiology, Tokyo Medical University Hospital, 6-7-1 Nishi-shinjuku, Shinjuku-ku, Tokyo 160-0023, Japan; Department of Radiology, Tokyo Medical University Hospital, 6-7-1 Nishi-shinjuku, Shinjuku-ku, Tokyo 160-0023, Japan; Department of Orthopedic Surgery, Tokyo Medical University Hospital, 6-7-1 Nishi-shinjuku, Shinjuku-ku, Tokyo 160-0023, Japan; Department of Anatomic Pathology, Tokyo Medical University Hospital, 6-7-1 Nishi-shinjuku, Shinjuku-ku, Tokyo 160-0023, Japan; Department of Anatomic Pathology, Tokyo Medical University Hospital, 6-7-1 Nishi-shinjuku, Shinjuku-ku, Tokyo 160-0023, Japan; Department of Radiology, Tokyo Medical University Hospital, 6-7-1 Nishi-shinjuku, Shinjuku-ku, Tokyo 160-0023, Japan; Department of Orthopedic Surgery, Tokyo Medical University Hospital, 6-7-1 Nishi-shinjuku, Shinjuku-ku, Tokyo 160-0023, Japan; Department of Anatomic Pathology, Tokyo Medical University Hospital, 6-7-1 Nishi-shinjuku, Shinjuku-ku, Tokyo 160-0023, Japan; Department of Radiology, Tokyo Medical University Hospital, 6-7-1 Nishi-shinjuku, Shinjuku-ku, Tokyo 160-0023, Japan

**Keywords:** Giant cell tumor of bone, rib tumor, chest wall tumor, H3.3 G34W, FDG-PET/CT

## Abstract

Giant cell tumor of bone (GCTB) rarely arises in the rib, and anterior rib involvement may mimic malignant chest-wall tumors. A 44-year-old woman presented with a palpable mass in the left anterior chest wall. Computed tomography (CT) revealed an osteolytic mass arising from the anterior left third rib, enlarging from 31 to 55 mm over ~2 months. Magnetic resonance imaging showed diffusion restriction, and fluorodeoxyglucose positron emission tomography/CT demonstrated intense uptake with a maximum standardized uptake value of 15.7, raising suspicion for malignancy. Needle biopsy suggested GCTB, but malignancy could not be excluded. Wide resection of the left second to fourth ribs was performed. Histology showed no sarcomatous transformation, and nuclear histone H3.3 with a glycine-to-tryptophan substitution at amino acid position 34 positivity in mononuclear tumor cells supported the diagnosis of GCTB. The patient remained recurrence-free for ~2 years. Anterior rib GCTB can mimic malignancy, requiring integrated radiological and pathological evaluation.

## Introduction

Giant cell tumor of bone (GCTB) is an intermediate, locally aggressive primary bone tumor composed of neoplastic mononuclear stromal cells and osteoclast-like multinucleated giant cells. According to the 2020 WHO Classification of Soft Tissue and Bone Tumours, GCTB typically affects the ends of long bones in the mature skeleton [1]. Rib involvement is rare, accounting for 5 of 213 cases (2.3%) in a major clinicopathological series of extra-craniofacial GCTB [2]. When GCTB occurs in the rib, it is usually located in the posterior aspect, whereas anterior rib involvement has been reported only rarely [3, 4]. GCTB of the rib may be difficult to distinguish from malignant bone tumors when rapid growth, cortical destruction, soft-tissue extension, or intense fluorodeoxyglucose uptake is present [5]. Histone H3.3 glycine-to-tryptophan substitution at amino acid position 34 (G34W) immunohistochemistry is useful for supporting the diagnosis of GCTB in morphologically challenging cases [6]. Here, we report a rare case of anterior rib GCTB that mimicked a malignant chest-wall tumor.

## Case report

A 44-year-old woman presented with a palpable mass in the left anterior chest wall. The lesion had initially been detected during a medical check-up, and breast cancer was suspected before further evaluation. Plain chest radiography showed a subtle opacity overlapping the left hilar region, but the origin and osseous involvement of the lesion were not clearly identified. Chest computed tomography (CT) demonstrated a soft-tissue mass arising from the anterior aspect of the left third rib with associated osteolytic bone destruction. The mass showed attenuation similar to that of skeletal muscle on non-contrast CT and gradual, mildly heterogeneous enhancement on contrast-enhanced CT. No obvious intratumoral calcification was observed. The mass displaced the pectoralis major muscle anteriorly without definite evidence of direct invasion and was in contact with the extrapleural fat adjacent to the pericardium. The maximum diameter measured on CT increased from 31 mm at the initial evaluation to 47 mm ~1 month later and to 55 mm ~2 months after the initial evaluation.

Magnetic resonance imaging showed a heterogeneous mass involving the left anterior chest wall. On T2-weighted imaging, the lesion showed signal intensity markedly higher than that of skeletal muscle but lower than that of cerebrospinal fluid. Diffusion-weighted imaging showed diffusion restriction, with apparent diffusion coefficient values of ~0.9–1.1 × 10^−3^ mm^2^/s. On T1-weighted imaging, the lesion was isointense to skeletal muscle, without an obvious fatty component or apparent high-signal area suggestive of hemorrhage. Fluorodeoxyglucose positron emission tomography/CT (FDG-PET/CT) demonstrated intense uptake within the lesion, with a maximum standardized uptake value (SUVmax) of 15.7. These findings raised suspicion for a malignant chest-wall tumor, including primary malignant bone tumor or metastatic disease (Fig. 1).

**Figure 1:**
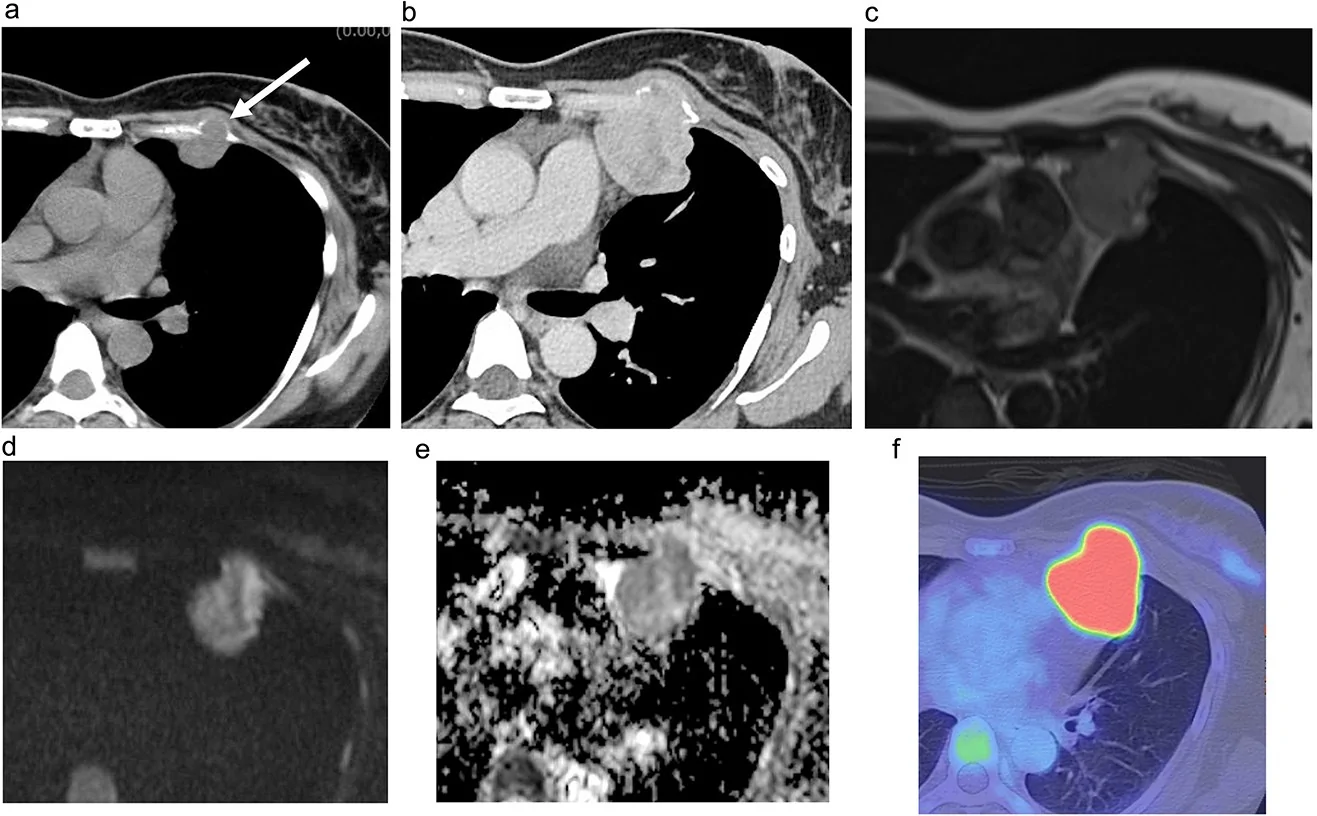
Radiological findings of the anterior left third-rib tumor. (a) initial CT image showing a 31-mm soft-tissue mass arising from the anterior aspect of the left third rib with osteolytic bone destruction (arrow); (b) contrast-enhanced CT image obtained ~2 months later showing enlargement of the mass to 55 mm, with mildly heterogeneous enhancement; the mass displaced the pectoralis major muscle anteriorly without definite invasion and was in contact with the extrapleural fat adjacent to the pericardium; (c) T2-weighted MR image showing signal intensity markedly higher than that of skeletal muscle but lower than that of cerebrospinal fluid; (d and e) diffusion-weighted MR image (*b* value = 800 s/mm^2^) and corresponding apparent diffusion coefficient map showing diffusion restriction, with high signal intensity on diffusion-weighted imaging and relatively low apparent diffusion coefficient values of ~0.9–1.1 × 10^−3^ mm^2^/s within the tumor; (f) FDG-PET/CT image showing intense FDG uptake, with an SUVmax of 15.7.

Needle biopsy revealed numerous multinucleated giant cells admixed with mononuclear spindle cells and hemorrhagic change. Scattered mitotic figures were observed; however, marked nuclear atypia and atypical mitotic figures were not identified. Although GCTB was suspected, malignancy could not be excluded preoperatively because of the rapid enlargement and aggressive imaging features.

Wide surgical resection was performed for diagnostic and therapeutic purposes. The tumor was resected en bloc with the left second to fourth ribs, and chest-wall reconstruction was performed using a metallic mesh covered with a pectoralis major muscle flap. Gross examination of the resected specimen showed a well-circumscribed, tan-white solid mass. Histologically, the tumor consisted of diffusely proliferating spindle-shaped mononuclear cells admixed with numerous osteoclast-like multinucleated giant cells. Foci of necrosis and hemorrhage were present. A few conventional mitotic figures were observed in the mononuclear tumor cells; however, marked nuclear atypia, atypical mitotic figures, and sarcomatous transformation were absent. The tumor showed irregular infiltration into the cortical bone and surrounding soft tissue. Immunohistochemistry demonstrated nuclear positivity for H3.3 G34W immunohistochemistry in the mononuclear tumor cells, whereas the osteoclast-like multinucleated giant cells were negative, supporting the final diagnosis of GCTB (Fig. 2). Although the tumor microscopically involved a focal portion of the surgical margin, no additional treatment was administered, and the patient was followed with serial imaging. The postoperative course was uneventful, and the patient was discharged 2 weeks after surgery. At ~2 years after surgery, she remained free from local recurrence or distant metastasis.

**Figure 2:**
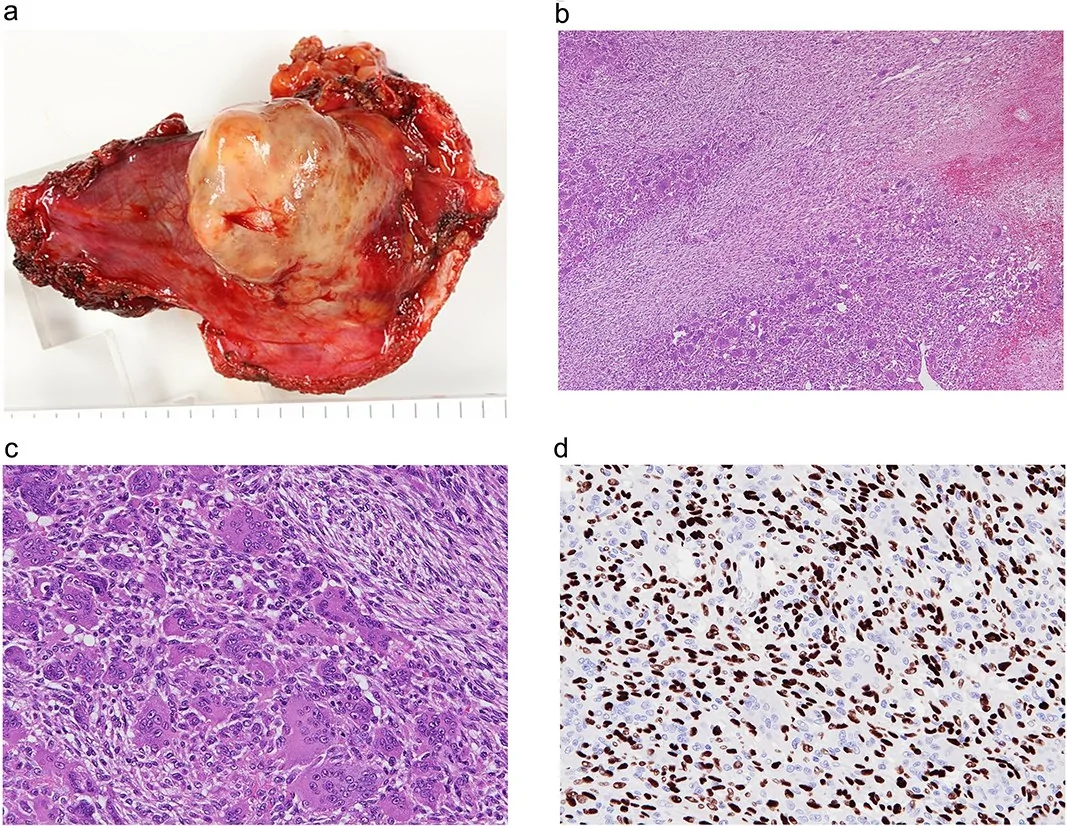
Gross and histopathological findings of the resected specimen. (a) gross appearance of the resected specimen showing a well-circumscribed, tan-white solid mass; (b and c) hematoxylin and eosin-stained photomicrographs showing focal necrosis and hemorrhage at low power (b) and spindle-shaped mononuclear tumor cells admixed with numerous osteoclast-like multinucleated giant cells without marked nuclear atypia at high power (c); (d) immunohistochemical staining for histone H3.3 G34W showing nuclear positivity in the mononuclear tumor cells but not in the osteoclast-like multinucleated giant cells, supporting the diagnosis of GCTB.

## Discussion

GCTB rarely arises in the rib, and anterior rib involvement is particularly uncommon. When GCTB occurs in the rib, it is usually located in the posterior aspect, whereas only a limited number of cases involving the anterior rib have been reported [3]. Anterior rib GCTB may present as a chest-wall mass and requires surgical resection because of its locally aggressive behavior and the difficulty of excluding malignancy preoperatively [4]. In the present case, rapid enlargement, osteolytic bone destruction, soft-tissue mass formation, diffusion restriction, and intense FDG uptake raised strong suspicion for a malignant chest-wall tumor.

High FDG uptake is not specific to malignancy. GCTB may show marked FDG accumulation, probably reflecting the metabolic activity of mononuclear stromal cells and osteoclast-like giant cells, and SUVmax alone may be insufficient to differentiate GCTB from malignant bone tumors [7]. Similarly, diffusion restriction and low apparent diffusion coefficient values can suggest high cellularity but may overlap between benign aggressive and malignant bone tumors. Therefore, the aggressive imaging features in this case should be interpreted as indicators of high biological activity rather than definitive evidence of malignancy.

Histopathological and immunohistochemical evaluation was essential for the final diagnosis. Although the tumor showed hemorrhage, necrosis, scattered mitotic figures, cortical destruction, and soft-tissue infiltration, marked nuclear atypia, atypical mitotic figures, and sarcomatous transformation were absent. Mutations in the H3 histone family member 3A (H3F3A) gene, which encodes histone H3.3, are characteristic molecular alterations in GCTB, and H3.3 G34W immunohistochemistry is useful for distinguishing GCTB from other giant cell-rich bone lesions [6]. In this case, H3.3 G34W-positive mononuclear cells supported the diagnosis of GCTB.

Wide resection was selected because malignancy could not be excluded preoperatively. Although the tumor focally involved the surgical margin, the patient remained recurrence-free for ~2 years without additional treatment. Nevertheless, continued long-term follow-up is warranted because local recurrence can occur after treatment of GCTB, particularly when complete local control is uncertain [8].

## Data Availability

Data sharing is not applicable to this article as no datasets were generated or analyzed during the current study.
